# Immunotherapy for a POLE Mutation Advanced Non-Small-Cell Lung Cancer Patient

**DOI:** 10.3389/fphar.2022.817265

**Published:** 2022-03-04

**Authors:** Yang Fu, Yue Zheng, Pei-Pei Wang, Yue-Yun Chen, Zhen-Yu Ding

**Affiliations:** Cancer Center, State Key Laboratory of Biotherapy, Department of Biotherapy, West China Hospital, West China Medical School, Sichuan University, Chengdu, China

**Keywords:** POLE mutation, lung adenocarcinoma, PD-L1, brain metastases, CR

## Abstract

Currently, the predictive role of POLE mutations for immunotherapy is under intense investigation. The POLE gene encodes one of the four subunits of DNA polymerase important for DNA replication and repair. POLE mutations are related to other favorable predicative factors such as high expression of PD-L1, high TMB, and infiltration of CD8^+^ cells in the tumor microenvironment. No formal clinical trials studied the efficacy of immunotherapy in lung patients harboring POLE mutation, and only few cases were mentioned in the literature. Moreover, lung cancer patients are prone to brain metastasis, which is notorious for the unresponsiveness to chemotherapy. The efficacy of immunotherapy for brain metastasis is still controversial. Here, we described a case of a POLE^mt^ non-small-cell lung cancer (NSCLC) patient with brain metastasis who was treated with immunotherapy. His brain lesions disappeared after treatment. Our report strongly supported the benefit of immune-combined therapy for advanced NSCLC patients with POLE mutation, even with brain metastasis.

## Case Report

**FIGURE 1:**
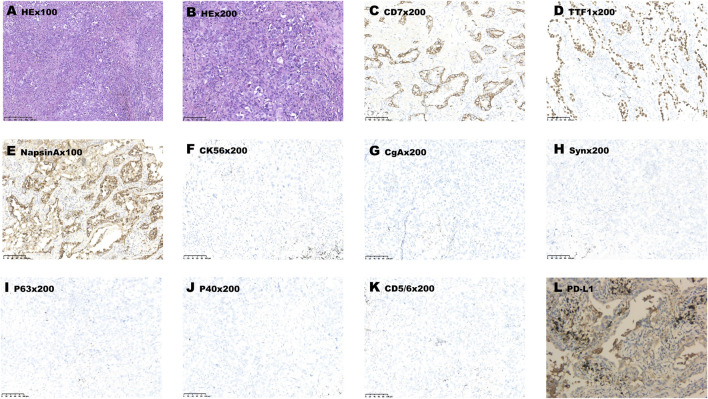
Pathological examination showed adenocarcinoma morphology **(A,B)**. **(C–L)** Immunohistochemistry data: CK7 (+), TTF-1 (+), NapsinA (+), CK5/6 (−), P63 (−), P40 (−), CD56 (−), CgA (−), Syn (−) and PD-L1 (+, positive proportion about 30%), supported the diagnosis. Original magnification: **(A)** ×100 and **(B–K)** × 200.

**FIGURE 2:**
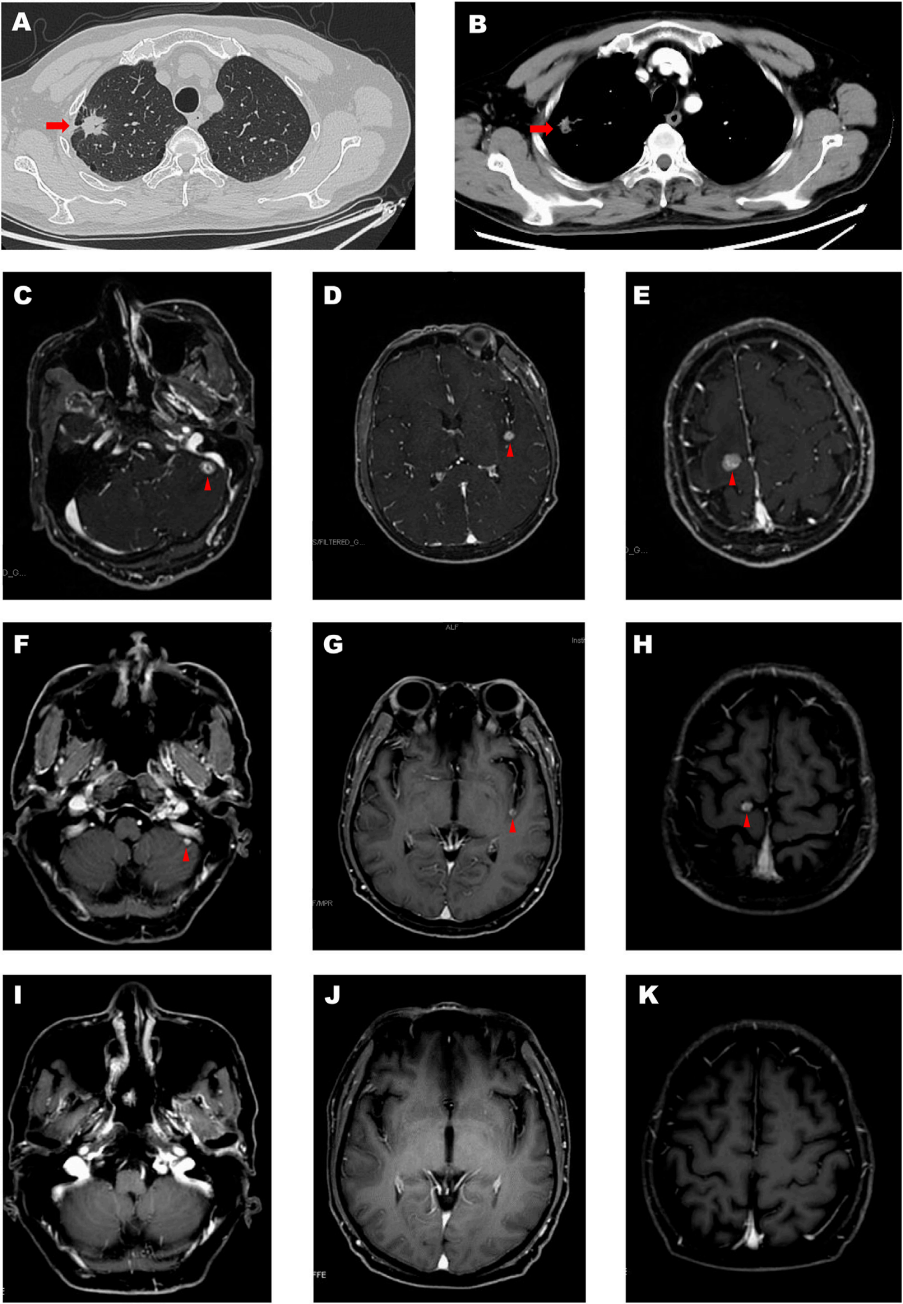
The red arrow represents the primary lesion of the lung **(A,B)**. The red triangle represents brain metastases **(C–H)**. And the enhanced MRI showed complete response of brain metastases **(I–K)**.

A 65-year-old man was admitted because of a nodule in the upper lobe of his right lung in his annual health screen without any discomfort. He had a smoking history of 30 years. The patient underwent an enhanced CT scan of his head before surgery, and no obvious metastases were found. He underwent right upper lobectomy and lymph node dissection. Postoperative pathological examination revealed invasive adenocarcinoma (alveolar, papillary, and solid) with pleural involvement, supported by typical immunohistochemistry (IHC) staining as follows: CK7(+), TTF-1 (+), NapsinA (+), CK5/6 (−), P63 (−), P40 (−), CD56 (−), CgA (−), Syn (−), and Ki-67 (50%). PD-L1 expression of the tumor proportion score (TPS) was evaluated using the IHC 22C3 pharmDx assay, and a combined positive sore of 30 was assessed (Figure 1). All the 13 excised lymph nodes were free of tumor cells including group 2 (0/2), group 4 (0/2), group 7 (0/3), and group 11 (0/1). He was diagnosed with pT2aN0M0, IB stage. However, after surgery, multiple enhanced intracranial nodules were observed in contrasted head MRI (Figures 2C–E) without neurological symptoms. The diagnosis was corrected to cT0N0M1c, IVc stage. The next-generation sequencing of his tumor detected POLE mutation (exon 26, p. P1025fs, 47.81%) and TP53 mutation (exon5, c.376-1G>A, 5.74%), microsatellite stabilization (MSS), and TMB 16.95 mut/Mb.

He was prescribed with two cycles of combination therapy of pemetrexed (500 mg/kg, Hansoh Inc.) plus carboplatin (AUC = 5, Yangtze Inc.) plus bevacizumab (7.5 mg/kg, Roche Inc.) plus tislelizumab (an anti-PD1 antibody, BeiGene Inc., 200 mg). After two cycles of therapy, the intracranial metastases became smaller in size (from 1.6 cm × 1.0 cm to 0.6 cm × 0.6 cm, Figures 2F–H). After four cycles of combined therapy, the metastases had completely disappeared (Figures 2I–K). He received tislelizumab, pemetrexed, and bevacizumab for two cycles of consolidation therapy. After six cycles of treatment, the patient felt fatigue and poor appetite. We tested the ACTH, 24 h urinary free cortisol excretion (UFC), and 8 h cortisol (PTC-8). The results showed that the 24 h UFC (4.8 ug/24h, normal: 20.3–127.6 ug/24h) and PTC-8 (19.9 nmol/L, normal: 133.0–537.0 nmol/L) decreased significantly. The patient was diagnosed with immune-related hypophysitis (grade 2) after multi-disciplinary treatment. He received glucocorticoids for a week, and the hypophysitis gradually relieved. Then, the treatment was switched to tislelizumab and bevacizumab for six cycles until now. Currently, 11 months after the initiation of the combined therapy, the patient is still on therapy and responding with no further treatment-related adverse events. The complete treatment process of the patient is shown in Table 1.

**TABLE 1:** Complete treatment process of the patient.

	The timetable of the entire disease process
September 2020	Annual health screen
November 2020	Right upper lobectomy and lymph node dissection
December 2020	Brain metastases
December 2020–February 2021	Two cycles of tislelizumab plus pemetrexed- carboplatin and Bev
February 2021	Partial response after two cycles of treatment
February 2021–April 2021	Two cycles of tislelizumab plus pemetrexed- carboplatin and Bev
April 2021	Complete response after four cycles of treatment
April 2021–June 2021	Two cycles of tislelizumab plus pemetrexed and Bev
June 2021	Complete response
June 2021	Immune-related hypophysitis (grade 2)
July 2021–November 2021	Six cycles of tislelizumab plus Bev
November 2021	Complete response

## Discussion

In the past decade, immune checkpoint inhibitors (ICIs) have emerged as a new treatment modality beyond chemotherapy for advanced non-small-cell lung cancer (NSCLC) without driver mutations. However, the question is that only a minority (20–30%) of patients can benefit from immunotherapy (Reck et al., 2016; Hellmann et al., 2019).

Currently, the predictive role of POLE mutations for immunotherapy is under intense investigation. The POLE gene is located in chromosome 12q24.33, encoding one of the four subunits of DNA polymerase important for DNA replication and repair (Rossi et al., 2020). POLE mutations are related to other favorable predicative factors such as high expression of PD-L1, high TMB, and infiltration of CD8^+^ cells in the tumor microenvironment (TME) (Wang et al., 2019; Yao et al., 2019). In one report, the density of CD8^+^ T cells was consistently higher in tumors harboring POLE mutation (POLE^mt^) than wild-type (POLE^wt^), either in endometrial cancer (59.4 vs. 24.7 CD8^+^ cells per HPF, *p* = 0.11) or colorectal intraepithelial neoplasia (59.4 vs. 14.8 CD8^+^ cells per HPF, *p* = 0.029) or colorectal cancer (154.9 vs. 34.0 CD8^+^ cells per HPF, *p* value undescribed) (Temko et al., 2018). The high CD8^+^ T-cell infiltration in POLE^mt^ colorectal cancer was also reported by Domingo et al. (Domingo et al., 2016). In POLE^mt^ endometrial cancer (*n* = 37), PD-L1 expression (>1%) was 29.6% and intratumoral T-cell infiltrates were 27.8% (Pasanen et al., 2020). In another report, Howitt et al. showed a PD-L1 expression (>10%) of 84% and a number of 32.8 CD8+TIL per HPF in POLE^mt^ endometrial cancer (Howitt et al., 2015).

The positive relationship between POLE^mt^ and immunotherapy was studied in a pancancer research study (Wang et al., 2019). Patients harboring POLE^mt^ were divided into the MSS group and MSI group, and the prognosis of the MSI group was better. However, in another phase II multicenter study where metastatic or unresectable colorectal cancer patients (*n* = 33) with dMMR/MSI-H or POLE^mt^ were enrolled, salvage (≥2 line) avelumab was prescribed. Unfortunately, all patients with POLE^mt^ (*n* = 3) had progressive disease in 2 months (Kim et al., 2020). Therefore, the role of POLE^mt^ is still controversial, and more studies are needed.

POLE mutations are common in endometrial cancer and colorectal cancer, but account for only about 3% of NSCLC (Wang et al., 2019; Song et al., 2018). It was reported that POLE^mt^ was a favorable prognostic factor in lung cancer (Liu et al., 2018). A study found a mutation rate of 2.8% (9/319) in NSCLC patients, and all were adenocarcinomas. The TMB of these patients was 12.2/Mb, higher than 7.8/Mb for the rest. None had MSI tumors. Seven patients were positive for CD8^+^ T cells, and five patients had a PD-L1 expression more than 25% (Song et al., 2018). No formal clinical trials studied the efficacy of immunotherapy in these patients, and only few cases were mentioned in the literature (Table 2) (Rizvi et al., 2015; Johanns et al., 2016; Gong et al., 2017; Song et al., 2018; Lee et al., 2019; Veneris et al., 2019; Zhu et al., 2020).

**TABLE 2:** Summary of case reports observing the efficacy of ICI in POLE mutation cancer.

Source	Tumor	Age	Ethnicity	Stage	Gene	PD-L1	TMB	MSS	Line	Therapy	PFS	Response	Death
Song et al., 2018	NSCLC	45	Asian	IIIB	POLE p.E468K	40%	N/A	Yes	Second	AC + Atezo	8 months	PR	No
Rizvi et al., 2015	NSCLC	N/A	N/A	N/A	POLE	N/A	N/A	N/A	N/A	Pembro	14 months	PR	No
Rizvi et al.	NSCLC	N/A	N/A	N/A	POLE	N/A	N/A	N/A	N/A	Pembro	10 months	PR	No
Zhu et al., 2020	Cervical carcinosarcoma	58	Asian	IV	POLE p.Pro286Arg, p.Ala724Val	N/A	691.3	Yes	Fourth	Pembro	11 months	CR	No
Johanns et al., 2016	Glioblastoma IV	31	N/A	IV	POLE R793C, V1002A	N/A	N/A	N/A	Second	Pembro	4 months	PR	No
Veneris et al., 2019	Endometrial cancer	49	N/A	IV	POLE c.1231GNT	10%	305.6	Yes	Second	Pembro	6 cycles	PR	No
Lee et al., 2019	Cervical cancer	55	N/A	IB3	POLE P286R	10%	N/A	Yes	Maintenance	Pembro	4 yrs	——	No
Gong et al., 2017	Colorectal cancer	81	Hispanic	IV	POLE V411L	100%	N/A	Yes	Third	Pembro	8 cycles	PR	No

NSCLC, non-small-cell lung cancer; AC, pemetrexed plus cisplatin; Atezo, atezolizumab; Pembro, pembrolizumab; yrs, years.

Moreover, lung cancer patients are prone to brain metastasis, which is notorious for unresponsiveness to chemotherapy. The efficacy of immunotherapy for brain metastasis is still controversial. In the brain metastasis subgroup of OAK research, atezolizumab outperformed docetaxel in the survival of these patients (16.0 vs. 11.9 months, *p* = 0.163, HR = 0.74) (Gadgeel et al., 2019). Recently, pembrolizumab was tested in a phase II trial for NSCLC with brain metastases, and 42 patients were divided into cohort 1 (PD-L1≥1%, *n* = 37) or 2 (PD-L1<1%, *n* = 5). The ORR of cohort 1 was 29.7% with four patients having CR, while there were no objective responses in cohort 2 (Goldberg et al., 2020). Another phase II trial evaluated the safety and efficacy of pembrolizumab on melanoma and NSCLC with brain metastases. All 18 patients in the NSCLC cohort had PD-L1 ≥ 1%. The ORR in this cohort was 33% (CR: *n* = 4, PR: *n* = 2), and the median survival was 7.7 months (Goldberg et al., 2016). In the RATIONALE 304 study, tislelizumab plus chemotherapy had a significantly longer median PFS than chemotherapy (9.7 vs. 7.6 m, *p* = 0.004). In addition, 18 NSCLC patients with brain metastases were enrolled in this study; however, it did not give exact data for the subgroup (Lu et al., 2021). Bevacizumab had encouraging efficacy against NSCLC with brain metastases. The combination of bevacizumab plus carboplatin and paclitaxel in the treatment of advanced non-squamous NSCLC was tested in phase II trial BRAIN (NCT00800202). The results showed that the ORR of intracranial lesions, median PFS, and median OS were 61.2%, 6.7 months, and 16.0 months, respectively (Besse et al., 2015). So, we chose four-drug combination therapy for this patient.

In conclusion, in this report, we described a case of a POLE^mt^ NSCLC patient with brain metastasis who was treated with immunotherapy plus chemotherapy and bevacizumab. His brain lesions disappeared after treatment. Our report strongly supported the benefit of immune-combined therapy for advanced NSCLC patients with POLE mutation, even with brain metastasis.

## Data Availability

The original contributions presented in the study are included in the article/Supplementary Material, further inquiries can be directed to the corresponding author.
